# Supplementary material for: On quantum gravity tests with composite particles
Source: Nat Commun. 2020 Aug 6;11:3900. doi: 10.1038/s41467-020-17518-5 (PMC7413341; doi:10.1038/s41467-020-17518-5)
Supplement: Supplementary file 1 — Supplementary Information [file 41467_2020_17518_MOESM1_ESM.pdf]

Reviewers' Comments:

Reviewer #1:
Remarks to the Author:
The paper by Kumar and Plenio discusses the possibility to test a class of quantum gravity theories in which a minimal length scale, possibly of the order of the Planck length, is obtained by means of deformed commutators.

In particular the paper focuses on the possibility to test these theories and obtain an upper bound in the deformation parameter \beta using composite macroscopic system made of many constituents.

I see three main results of the paper:

i) a new phenomenological proposal for comparing this kind of tests introducing a phenomenological dependence upon the number of particles N, in order to take into account the so-called "soccer-ball" problem. In this proposal \beta is replaced by \beta/N^\alpha, and one has to compare results in a 2d plane for \beta and \alpha.
ii) the use of an accurate old experiment testing the amplitude dependence of the period of a pendulum for deriving a new more powerful bound for the deformation parameter and \alpha, improving recent bounds derived from experiments with various microscopic and macroscopic resonators.
iii) The analytical study of the deformed algebra and the demonstration of the equivalence of the classical treatment with deformed Poisson parentheses and a quantum treatment with deformed commutator and appropriately defined deformed coherent state.

The paper is clearly written, and the results are novel and interesting, implying that the paper certainly deserves publication. However I think that, even though the topic is interesting and up-to-date, the above results are of quite technical nature and therefore not suitable for the broad and general audience of Nature Communications. I think it would be more suitable for a more technical physics journal.

To be more specific I add some comments on the three above results.

1) Proposing to include an additional scaling of the deformation parameter upon the number of constituents adds a second phenomenological parameter (\alpha).
In principle such a scaling should be deduced from theory and there are even arguments (see eg ref. 19) for 1/N^2 or 1/N scalings. Moreover the results of Ref 5, at three different masses suggest a scaling close to this. Nonetheless the difficulty of dealing with composite systems is here bypassed by adding this second exponent parameter \alpha. This is certainly legitimate in these phenomenological approaches, but the meaning of this approach and of \alpha remains quite unclear and a better justification would be appreciated.

2) The pendulum experiment used by the authors is able to provide a much better bound because in this case the nonlinearity is well known and measurable. In this respect, the limiting factor of Ref 5 and 8 is that the measured nonlinearities cannot be compared with known analytical models and therefore in those cases they cannot take into account the intrinsic nonlinearities of the systems adopted.

Moreover I am quite skeptical about the improved estimation for the magnetically levitated nanosphere. It is quite unrealistic and even not too much correct to assume the limit on the trapping frequency only given by the extremely low value of the nanoparticle damping. In fact, in this cases, the drifts and phase/frequency noise of the resonance frequency cannot be neglected and it usually becomes the main source of error in the estimation of the resonance frequency. Therefore I would take a much more conservative value for the frequency error. THis frequency

noise is the analogous for qubit systems of T_2 dephasing processes, while mechanical damping refers to energy relaxation processes, T_1, which may be typically longer.

3) I have found the comparison with the classical approach with Poisson parentheses and the quantum one with the commutator very interesting. As correctly pointed out in the paper, this works with a given (well justified) choice of coherent state, and I wonder if this approach con be further generalized and make it independent of the coherent state definition.

Reviewer #2:
Remarks to the Author:
Rather than offering a recommendation I prefer to provide to the editors my assessment of the main strengths and the main weaknesses of this study, hoping to be of assistance for determining whether the manuscript is suitable for NatComm

Overall I believe that this is valuable exploratory research. It proposes a way to face the challenge exposed in Ref.[19]
(and partly in Ref.[3]) concerning the use of macroscopic systems in quantum-gravity phenomenology. The authors propose to handle this by modeling the dependence of the effects on the macroscopicity of the system, which in principle is a solid approach.

The main limitation of the analysis resides in the assumption
that the magnitude of the effects should scale simply like
the inverse of some (fractional) power of the number of
constituents of a macroscopic system. This might be a good (though still rough) starting point for phenomenology based on weakly interacting constituents, but in a typical macroscopic body the constituents interact strongly.
When interactions are important one may expect that two different bodies, even when composed by the same number of constituents, would be affected by effects of different magnitude.

Reviewer #3:
Remarks to the Author:
The paper is very well written and treats a topic that is timely and definitely of interest to the scientific community: how can we use low-energy physics (not a particle accelerator) in a lab to potentially devise bounds on possible modifications to quantum physics due to influences from quantum gravitational effects. In particular, the authors consider changes to the commutation relation between the position and momentum operators. They argue that the commutation relation should depend on two yet unknown parameters, which one can put bounds on by doing the proper experiments. The authors demonstrate this by analyzing existing experimental data to determine what bounds these experiments put on the parameters of their model. They also suggest how to possibly improve on existing experiments in order to achieve more stringent bounds. I found the paper a very interesting read, and given that I am also working on test of the foundations of quantum physics, I can confirm that the authors' research definitely is of interest for the field and should have a notable impact. I therefore recommend the paper to be accepted for publication in Nature Communications, possibly with a few minor modifications given some small comments I will list below:

.) for equation 10, the authors assume that β is much smaller than 1. If my estimations are correct based on the parameters the authors provide, then this should definitely be true for α larger than 0.01, but for smaller values of α, β tends towards larger values. Nevertheless, I get the impression that it never rises above a few percent, so it is probably true that $\beta \ll 1$ can be seen as valid for all values of α. It might still be worth a short

comment in the manuscript, because it was not immediately clear to me that this assumption would hold true.

.) the authors specify $\gamma=1.2\times 10^{-7}\,$Hz and say that $\Delta\omega\simle\gamma/\sqrt{M}$, but γ is in Hz while I expect ω is in rad/s -> is there a factor of 2π missing? Since this corresponds to nearly an order of magnitude, it might be relevant for the bound the authors set.

.) if I take the values the authors state beneath equation (16) in combination with their equations (16) and (15), and if I assume (as the authors state) $\omega=\gamma/\sqrt{M}$, I arrive at an angular frequency of $\omega=36.8148\,$rad/s, a mass of 80.8437kg and a lower bound on α that is 0.428224 - compared to the lower bound of 0.35 the authors state. Did I make a mistake or did they? If one assumes that the bound on $\Delta\omega$ still needs to be multiplied by 2π, then I get a lower bound of 0.398512 for α - which still differs from the value the authors give. I wanted to point this out in case my calculations were correct.

.) equation (18): it is not so obvious to me that an increase of mass helps. In the exponent, one has a negative term proportional to mass AND proportional to N^2_p and a positive term proportional to N_p and to the average momentum. An increase in mass will lead to a VERY strong change in the negative term that does not depend on the average momentum, and it will also lead to an increase in the term that does depend on the average momentum squared. The latter term depends on the mass to the third power, the former term depends on the mass to the fourth power. In addition, there is a λ dependence, which may also depend on the test mass. The authors actually mention that large masses may reduce the coupling. Nevertheless, the precise parameter dependence would be interesting to look into in more detail.

.) APPENDIX, subsection 3 - the authors introduce Gazeau-Klauder coherent states $\vert J,\gamma\rangle$ - I think it would be useful for a more intuitive understanding to describe what meanings J and γ have in this respect.

Reviewer #4:
Remarks to the Author:
This is an excellent theoretical/experimental article which merits publication in this journal. However, I have a couple of issues with it, which I hope the authors can address before a final version is published.

Above all I think that the article mixes very different matters, which indeed may be related, but this should be spelled out. Usually the issues of the "soccer ball problem" and of the dependency of deformations on the number of particles, N, appear firstly in momentum space. It is found that dispersion relations should depend on N if they are to avoid producing obvious contradictions (such as a maximal mass/energy for macroscopic systems). Here, instead, the dependence appears in the commutation relations between position and momentum space.
However, it is well known that in order to render deformed dispersion relations observer invariant one should impose non-linear "Lorentz" transformations to energy and momentum.
In turn this creates problems in defining their dual space of positions. It has been suggested that the factorization of duals cannot even be achieved in an invariant way, in the face of this situation (a nice split of duals requires linearity of transformations). For example it may be necessary that the position transformation rules of a particle (or system of particles) depend on its energy and momentum.

Hence I am puzzled at this mixture of x and p in a scenario where clearly p is already modified in a form that depends on N, and this affects the 'x'. The authors should explain how their work relates in detail to previous definitions of the "soccer ball problem", typically hailing from momentum

space alone, with consequences to position space which are highly non-trivial. Is there a "rainbow metric", which would also be N dependent, besides energy dependent? In what way is the Poisson bracket
postulated here consistent with the relativity of inertial frames
(assuming it is)? The fact that the system considered is non-relativistic is no excuse for skirting these problems.

In addition, I note that the appearance of N in the dispersion relations is usually related to the issue of how to add energies and momenta. Often solving the soccer ball problem implies non-associative addition laws (since the grand-total must know how many "terms" there are in the "sum", thereby precluding "associating" them). I doubt a detailed analysis of the inner workings of the experiments reported in this paper could be altogether insensitive to this issue.

Having said this, I hope that the authors can address these matters so that I can recommend this paper for publication.

Response to Referee 1

> The paper by Kumar and Plenio discusses the possibility to test a class of quantum gravity theories in which a minimal length scale, possibly of the order of the Planck length, is obtained by means of deformed commutators. In particular the paper focuses on the possibility to test these theories and obtain an upper bound in the deformation parameter β using composite macroscopic system made of many constituents.
>
> I see three main results of the paper:
>
> i) a new phenomenological proposal for comparing this kind of tests introducing a phenomenological dependence upon the number of particles N, in order to take into account the so-called "soccer-ball" problem. In this proposal β is replaced by β/N^{α}, and one has to compare results in a 2d plane for β and α.
>
> ii) the use of an accurate old experiment testing the amplitude dependence of the period of a pendulum for deriving a new more powerful bound for the deformation parameter and α, improving recent bounds derived from experiments with various microscopic and macroscopic resonators.
>
> iii) The analytical study of the deformed algebra and the demonstration of the equivalence of the classical treatment with deformed Poisson parentheses and a quantum treatment with deformed commutator and appropriately defined deformed coherent state.

We agree with the complete and accurate summary of the manuscript provided by the referee.

> The paper is clearly written, and the results are novel and interesting, implying that the paper certainly deserves publication.

We thank the referee for the positive assessment of the novelty and clarity of presentation of the manuscript.

> However I think that, even though the topic is interesting and up-to-date, the above results are of quite technical nature and therefore not suitable for the

broad and general audience of Nature Communications. I think it would be more suitable for a more technical physics journal.

As mentioned by the referee, our manuscript makes three main contributions, namely the introduction of a new formalism followed by the experimental test and rigorous justification of this formalism. These contributions, only when put together, enable rigorous tests that use composite particles to obtain meaningful bounds on quantum gravity parameters.

First, experimental tests of quantum gravity are of interest to every physicist as it is one of the fundamental open problem in modern physics. Secondly, our proposed formalism is essential to all recently proposed tabletop tests of quantum gravity because these rely on composite systems to test deformed commutators. Because of its interest to a general audience and significance to a specialist in the field, we are confident that our work is suitable for publication in Nature Communications.

Nonetheless, as described in this response, we have made numerous improvements to the overall presentation of the manuscript in order to make it more broadly accessible. More specifically related to the broad and general interest, we have added text to the introduction of the manuscript to firstly connect the work with the soccer ball problem and secondly to further clarify the motivation behind the new formalism that we provide in this article. These changes are detailed in the responses to the other referees (i.e., the responses to Referees 4 and 2 respectively) and also highlighted in the appended updated manuscript. We trust that these changes further address the concerns of the referee.

To be more specific I add some comments on the three above results.

1) Proposing to include an additional scaling of the deformation parameter upon the number of constituents adds a second phenomenological parameter (α). In principle such a scaling should be deduced from theory and there are even arguments (see eg ref. 19) for $1/N^2$ or $1/N$ scalings. Moreover the results of Ref 5, at three different masses suggest a scaling close to this. Nonetheless the difficulty of dealing with composite systems is here bypassed by adding this second exponent parameter α. This is certainly legitimate in these phenomenological approaches, but the meaning of this approach and of α remains quite unclear and a better justification would be appreciated.

We thank the referee for this opportunity to clarify the meaning and relevance of the parameter α in our manuscript. The parameter α is mainly a tool to include

possible particle-number dependent corrections to the expected deformed canonical commutation relations. We would like to stress that the arguments in Ref. [19] suggest that the scaling with number of particles is $1/N$ or $1/N^2$ holds for quasi-rigid macroscopic bodies whose centre-of-mass degrees of freedom decouple from the degrees of freedom of individual constituents. This could hold for systems such as rigid solids at low temperatures, but might not be true in general. In fact, Ref. [19] states that the $1/N$ or $1/N^2$ scaling may not hold and it is important to model such bodies rather than assume some fixed scaling in the discussions section:

> "In such cases, the arguments I here reported would be inapplicable, but of course this does not mean that some naive guess work is then allowed. One should handle the tough challenge of modeling such bodies and figure out under which conditions the Planck-scale effects could be tangible."

We have added the following text in the manuscript to clarify this point.

> The reason for considering such a polynomial scaling with number of particles N (with exponent α) is the expected powerlaw dependence in quasi-rigid macroscopic bodies, i.e., those whose centre-of-mass momenta are the sum of constituents' individual momenta [19]. Once phenomenological evidence of deformed commutator models begins to accumulate, more refined models can be considered accounting for differences in the exact nature of commutator deformation experienced by bodies with different nature of interaction.

> 2) The pendulum experiment used by the authors is able to provide a much better bound because in this case the nonlinearity is well known and measurable. In this respect, the limiting factor of Ref 5 and 8 is that the measured nonlinearities cannot be compared with known analytical models and therefore in those cases they cannot take into account the intrinsic nonlinearities of the systems adopted.

We thank the referee for raising this point, which further clarifies the advantage of using massive pendula instead of opto-mechanical systems [3], micro- and meso-scale oscillators [5,8]. Based on the referee's suggestion, we have clarified this point in the manuscript:

> We note that such tight bounds were possible in our work as compared to those of previous works because the nonlinearities in a pendulum can

be computed precisely. In other systems, the intrinsic nonlinearities are unknown and therefore contribute additional uncertainties that lead to larger bounds on quantum gravity parameters.

Moreover I am quite skeptical about the improved estimation for the magnetically levitated nanosphere. It is quite unrealistic and even not too much correct to assume the limit on the trapping frequency only given by the extremely low value of the nanoparticle damping. In fact, in this cases, the drifts and phase/frequency noise of the resonance frequency cannot be neglected and it usually becomes the main source of error in the estimation of the resonance frequency. Therefore I would take a much more conservative value for the frequency error. This frequency noise is the analogous for qubit systems of T_2 dephasing processes, while mechanical damping refers to energy relaxation processes, T_1, which may be typically longer.

We thank the referee for raising this point, which we agree with. We understand that the more realistic error in frequency might be a few orders of magnitude larger, but since it is difficult to quantify with any degree of certainty, we simply increase it by several orders of magnitude for a more realistic bound on α. We have edited the section to now take a much more conservative and therefore more realistic parameters into account and also updated the related figure (FIG 1) in the manuscript.

However, we realise that such estimates are based on optimistic assessments of all the involved parameters. More realistically, the error in the frequency might not be limited by damping alone and the different parameters feasible in a single experiment might be somewhat suboptimal once all components are integrated together. Thus, we can conservatively assume that the actual precision in frequency is three orders of magnitude worse than the optimistic value suggested above, i.e., $\Delta\omega \lesssim 10^{-4}$ Hz, we obtain the bounds $\alpha > 0.24$ for $\beta_0 = 1$.

Furthermore, we have modified the figure caption to emphasise that the bounds are based on optimistic parameters.

The proposal to use massive levitated diamagnetic objects promises significant improvement in bounds if the optimistic parameters required for such an experiment can be obtained.

---

3) I have found the comparison with the classical approach with Poisson

parentheses and the quantum one with the commutator very interesting. As correctly pointed out in the paper, this works with a given (well justified) choice of coherent state, and I wonder if this approach con be further generalized and make it independent of the coherent state definition.

We appreciate this suggestion to make the analysis independent of the coherent state definition. This is indeed a very important open problem that needs to be addressed in a subsequent work but goes well beyond the scope of the present manuscript and would also not be accessible to the general reader. We had tried to solve a simplified variation of this problem, that is if the initial state is a thermal state. However, that turned out to be unclear because it was not possible to come up with a definition of a thermal state in this formalism of commutators being deformed as the displacement operator is not well defined. Other papers that have approached this problem have not successfully managed to match the classical and quantum descriptions so far.

First, we have added a full-column subsection clarifying the meaning of the coherent states that we employ in our analysis. This material is in Sec. IV B 3. Furthermore, the following text in the discussion section already mentions the difficulty in moving beyond the definition of coherent states used here.

> Another important open problem is to go beyond coherent states as the initial states to thermal states. Our analysis can straightforwardly be applied to thermal states in principle, but the definition of a thermal state under the deformed commutators is unclear.

Response to Referee 2

> Rather than offering a recommendation I prefer to provide to the editors my assessment of the main strengths and the main weaknesses of this study, hoping to be of assistance for determining whether the manuscript is suitable for NatComm.
>
> Overall I believe that this is valuable exploratory research. It proposes a way to face the challenge exposed in Ref.[19] (and partly in Ref.[3]) concerning the use of macroscopic systems in quantum-gravity phenomenology. The authors propose to handle this by modelling the dependence of the effects on the macroscopicity of the system, which in principle is a solid approach.

We agree with the referee's phrasing of the challenge addressed in this work, and we appreciate the positive comments of the referee regarding the value of our manuscript

in presenting solid exploratory research.

> The main limitation of the analysis resides in the assumption that the magnitude of the effects should scale simply like the inverse of some (fractional) power of the number of constituents of a macroscopic system. This might be a good (though still rough) starting point for phenomenology based on weakly interacting constituents, but in a typical macroscopic body the constituents interact strongly.

Here the referee highlights one of the assumptions made in our analysis, i.e., that of a polynomial scaling with respect to N. This assumption is reasonable for weakly interacting systems as pointed out by the referee. In fact, even for the case of typical macroscopic objects whose constituents interact strongly (for example solids at low temperatures), this assumption has been derived in Ref. [19]. In particular, Ref. [19] considers quasi-rigid particles whose centre-of-mass momenta are the sum of constituents' individual momenta. This includes macroscopic objects whose center-of-mass degrees are decoupled from other degrees and whose constituents have the same mass as each other, which is the case referred to by the referee. In such a situation, it is found that the deformations see a correction that scales polynomially with the number of particles. We have clarified this point in the manuscript by adding this new text:

> The reason for considering such a polynomial scaling with number of particles N (with exponent α) is the expected polynomial dependence in quasi-rigid macroscopic bodies, i.e., those whose centre-of-mass momenta are the sum of constituents' individual momenta [19].

> When interactions are important one may expect that two different bodies, even when composed by the same number of constituents, would be affected by effects of different magnitude.

We agree with the referee on this point that bodies in different regimes (for example in terms of energies of constituent particles or the nature of interaction) could have different scalings. Our formalism then also provides an avenue for obtaining preliminary information about these differences. For instance, if the applicable values of α are different for different bodies, then one could refine the model to consider this. We have added the following text to the manuscript to highlight this point.

> Once phenomenological evidence of deformed commutator models begins to accumulate, more refined models can be considered accounting for

differences in the exact nature of commutator deformation experienced by bodies with different nature of interaction.

Finally, we would also like to point out that the proposals put forth thus far have assumed that the parameters do not scale with N, i.e., $\alpha = 0$. Our manuscript goes beyond such unsubstantiated assumptions and shows that, based on experimental evidence, these simply cannot be true.

Response to Referee 3

> The paper is very well written and treats a topic that is timely and definitely of interest to the scientific community: how can we use low-energy physics (not a particle accelerator) in a lab to potentially devise bounds on possible modifications to quantum physics due to influences from quantum gravitational effects. In particular, the authors consider changes to the commutation relation between the position and momentum operators. They argue that the commutation relation should depend on two yet unknown parameters, which one can put bounds on by doing the proper experiments. The authors demonstrate this by analyzing existing experimental data to determine what bounds these experiments put on the parameters of their model. They also suggest how to possibly improve on existing experiments in order to achieve more stringent bounds.

We agree with the accurate summary of the manuscript provided by the referee.

> I found the paper a very interesting read, and given that I am also working on test of the foundations of quantum physics, I can confirm that the authors' research definitely is of interest for the field and should have a notable impact. I therefore recommend the paper to be accepted for publication in Nature Communications, possibly with a few minor modifications given some small comments I will list below:

We appreciate the referee's positive assessment of the manuscript.

> 1.) for equation 10, the authors assume that β is much smaller than 1. If my estimations are correct based on the parameters the authors provide, then this should definitely be true for α larger than 0.01, but for smaller values of α, β tends towards larger values. Nevertheless, I get the impression that it never rises above a few percent, so it is probably true that $\beta \ll 1$ can be seen

as valid for all values of α. It might still be worth a short comment in the manuscript, because it was not immediately clear to me that this assumption would hold true.

We thank the referee for this helpful suggestion that would clarify the manuscript. We have added the following text to the manuscript:

> Here, we have calculated the correction to the time period to first order in the quantum gravity parameter β. We argue that if β were not, in fact, small, then a much larger deviation in the time period would be observed. In such a case, more detailed calculations would be required to obtain accurate bounds. We show that the time period of a pendulum obtained from experimental data in Sec. II B corroborates this assumption.

2.) the authors specify $\gamma = 1.2 \times 10^{-7}$ Hz and say that $\Delta\omega \lesssim \gamma/\sqrt{M}$, but γ is in Hz while I expect ω is in rad/s $\rightarrow$ is there a factor of 2π missing? Since this corresponds to nearly an order of magnitude, it might be relevant for the bound the authors set.

We thank the referee for going over the calculations in detail. We would like to note that in all our calculations, we follow SI units, so the frequency ω is reported in Hz. In other words, there is no factor of 2π missing. We have added the following text to clarify the units used in this calculation.

> This leads to the frequency of oscillations $\omega = 36.71$ Hz, from which we estimate that for $\beta_0 = 1$, we obtain $\alpha > 0.35$ for a single measurement $N_m = 1$.

3.) if I take the values the authors state beneath equation (16) in combination with their equations (16) and (15), and if I assume (as the authors state) $\omega = \gamma/\sqrt{M}$, I arrive at an angular frequency of $\omega = 36.8148$ rad/s, a mass of 80.8437kg and a lower bound on α that is 0.428224 - compared to the lower bound of 0.35 the authors state. Did I make a mistake or did they? If one assumes that the bound on $\Delta\omega$ still needs to be multiplied by 2π, then I get a lower bound of 0.398512 for α - which still differs from the value the authors give. I wanted to point this out in case my calculations were correct.

We thank the referee for the careful reading and checking of calculations of the manuscript. The source of confusion here is the notation where M stands for the

number of measurements and m is the mass of the oscillator. Considering the correct variables, we were able to validate the results in the manuscript. We have changed this potentially confusing notation M to a more unambiguous N_m in the current revision as follows:

> If, in the experiment, no deviation from the expected frequency is observed, then $\Delta\omega \lesssim \gamma/\sqrt{N_m}$ where N_m is the number of measurements taken.

> 4.) equation (18): it is not so obvious to me that an increase of mass helps. In the exponent, one has a negative term proportional to mass AND proportional to N_p^2 and a positive term proportional to N_p and to the average momentum. An increase in mass will lead to a VERY strong change in the negative term that does not depend on the average momentum, and it will also lead to an increase in the term that does depend on the average momentum squared. The latter term depends on the mass to the third power, the former term depends on the mass to the fourth power. In addition, there is a λ dependence, which may also depend on the test mass. The authors actually mention that large masses may reduce the coupling. Nevertheless, the precise parameter dependence would be interesting to look into in more detail.

The referee claims that in Eq. (18), increasing the mass leads to a stronger change in the second term than in the third term of the phase. This would be true if N_p were the number of particles in the system. However, N_p stands for the average number of photons in the light pulse, as mentioned in the text below Eq. (17).

About the precise parameter dependence on the mass, we thank the referee for this helpful suggestion. We agree with the referee and have added the following paragraph in the manuscript to clarify this point.

> To understand better the mass dependence of the quantum gravity terms, we consider the mass dependence of λ using an established model [3]
>
> $$\lambda = \frac{4\mathcal{F}}{\lambda_L}\sqrt{\frac{\hbar}{m\omega}} \quad (1)$$
>
> where $\mathcal{F}$ is the finesse of the cavity and λ_L is the wavelength of light used. With this scaling of λ with the mass m, we see that the first negative term scales as $\frac{1}{m}$ while the second one scales as m. Hence, we see that the extra term arising from a non-zero initial momentum can, in principle, be made large by choosing a more massive oscillator.

> 5.) APPENDIX, subsection 3 - the authors introduce Gazeau-Klauder coherent states $|J,\gamma\rangle$ - I think it would be useful for a more intuitive understanding to describe what meanings J and γ have in this respect.

We thank the referee for this suggestion. We agree that an introduction to these states would be valuable to the reader. In light of this, we have added a full one-column long subsection (Sec. IV B 3) to describe the meanings of J and γ. In this section, we have described the properties satisfied by the Gazeau-Klauder states and shown that if the system is a harmonic oscillator, the Gazeau-Klauder state for such a system reduces to a coherent state $|\xi\rangle$ where $\xi = \sqrt{J}\mathrm{e}^{-i\gamma}$.

Response to Referee 4

> This is an excellent theoretical/experimental article which merits publication in this journal.

We thank the referee for the positive assessment of the manuscript.

> However, I have a couple of issues with it, which I hope the authors can address before a final version is published.
>
> Above all I think that the article mixes very different matters, which indeed may be related, but this should be spelled out. Usually the issues of the "soccer ball problem" and of the dependency of deformations on the number of particles, N, appear firstly in momentum space. It is found that dispersion relations should depend on N if they are to avoid producing obvious contradictions (such as a maximal mass/energy for macroscopic systems). Here, instead, the dependence appears in the commutation relations between position and momentum space. However, it is well known that in order to render deformed dispersion relations observer invariant one should impose non-linear "Lorentz" transformations to energy and momentum. In turn this creates problems in defining their dual space of positions. It has been suggested that the factorization of duals cannot even be achieved in an invariant way, in the face of this situation (a nice split of duals requires linearity of transformations). For example it may be necessary that the position transformation rules of a particle (or system of particles) depend on its energy and momentum.

The reviewer refers to a formulation of quantum gravity where the laws of conservation

of momentum are deformed by Planck-scale dependent corrections. This momentum space is dual to Lie-algebra spacetimes [Phys. Rev. D 84, 084010 (2011)], i.e., different position coordinates no longer commute, but their commutator picks up a Planck-scale-dependent contribution as

$$[x^m, x^n] = i\ell\theta_k^{mn} x^k. \tag{2}$$

Indeed, this formalism of quantum gravity has pleasing properties, including Lorentz invariance, and it is in this formalism that the soccer-ball problem is apparent.

We would like to note that in the current work, our manuscript considers a different formulation of quantum gravity, namely that of deformed position and momentum commutators:

$$[x, p_x] = i\hbar\left(1 + \frac{\beta_0}{(M_p c)^2} p_x^2\right). \tag{3}$$

This formulation postulates that the existence of a minimal length scale can be considered to be a result of deformed position and momentum commutators. This has been postulated and studied in, for instance, Refs. [9,10,11,13,15]. As pointed out by the referee, this formalism does not necessarily obey Lorentz invariance but it opens the possibility of tabletop experimental tests such as Refs. [3,5,6,7,8,18]. These tabletop experiments inform our motivation to use the $[x, p_x]$ formalism rather than the momentum-space deformation.

To clarify our motivation, we have added the following text in the manuscript:

> Furthermore, we note that other models of quantum gravity could be considered, for instance, those involving non-commuting spacetime coordinates. However, our focus is on deformed position and momentum commutators because these underlie proposed tabletop tests of quantum gravity.

> Hence I am puzzled at this mixture of x and p in a scenario where clearly p is already modified in a form that depends on N, and this affects the 'x'. The authors should explain how their work relates in detail to previous definitions of the "soccer ball problem", typically hailing from momentum space alone, with consequences to position space which are highly non-trivial.

Since we are considering a different formalism of quantum gravity from the one alluded to by the reviewer, we understand that the phrase soccer-ball problem, as used in our manuscript, could have caused confusion. We have edited the sentence to now clarify that we are using a different formulation as follows:

Studies of the soccer-ball problem [21–23], which arises in a different framework of quantum gravity, also point toward a suppression of the Planck-scale corrections with number of particles.

Is there a "rainbow metric", which would also be N dependent, besides energy dependent? In what way is the Poisson bracket postulated here consistent with the relativity of inertial frames (assuming it is)? The fact that the system considered is non-relativistic is no excuse for skirting these problems.

As described above, unlike the momentum-space deformation formalism, our deformed commutator (or Poisson bracket) approach considered here is not consistent with the relativity of inertial frames. That said, the deformed commutator approach is grounded in the rigorous foundation set by Refs. [9,10,11,13,15] and exploited in proposed tabletop experiments of Refs. [3,5,6,7,8,18].

In addition, I note that the appearance of N in the dispersion relations is usually related to the issue of how to add energies and momenta. Often solving the soccer ball problem implies non-associative addition laws (since the grand-total must know how many "terms" there are in the "sum", thereby precluding "associating" them). I doubt a detailed analysis of the inner workings of the experiments reported in this paper could be altogether insensitive to this issue.

Here the referee points to the fact that in the momentum-space deformation formalism, the momenta of individual constituents add non-associatively, especially in the context of momentum conservation. Our deformed commutator approach also demonstrates a version of non-associative addition but in the context of deformed commutators, as evidenced by Eqs. (3) and (8) of Ref. [19]. Since the proposed tabletop experiments focus on measuring commutator deformations, we introduce the N^α term in the deformation to account for this non-associativity and propose to bound α. As mentioned in the responses to other reviewers, we have added text to clarify the meaning of α in context of the non-associativity of the deformations

The reason for considering such a polynomial scaling with number of particles N (with exponent α) is the expected polynomial dependence in quasi-rigid macroscopic bodies, i.e., those whose centre-of-mass momenta are the sum of constituents' individual momenta [19]. Once phenomenological evidence of deformed commutator models begins to accumulate, more refined models can be considered accounting for differences in the exact nature of commutator deformation experienced by bodies with different

nature of interaction.

Having said this, I hope that the authors can address these matters so that I can recommend this paper for publication.

The points raised by the referee are important problems in the field of quantum gravity. We hope we have addressed the concerns of the referee and clarified the manuscript.

The revised manuscript highlighting all additions and deletions is appended below. We trust that these modifications address the comments of the referees in full and hope that now the manuscript is suitable for publication in Nature Communications.

Reviewers' Comments:

Reviewer #1:
Remarks to the Author:
The authors have addressed all the issues I have raised in my first report and I think that the paper has improved in this respect. The approach and in particular the introduction of the exponent parameter \alpha remains phenomenological, and deeper arguments on why one should adopt such a more general parametrization of the deformation parameter are not provided. Despite the significant improvement of the presentation, I still think that the paper should be more suitable for a more technical journal.

Reviewer #2:
Remarks to the Author:

The authors have used my feedback and the feedback of other referees
to significantly improve the manuscript.
The model remains crude, but it is true that it is an attempt
to model something that noone else has tried to model and needs
to be modeled.
The improvements made to the manuscript are such that now this
crude model can me more efficiently received by the community.
In summary I don't think this model will guide the relevant sub-area
of quantum-gravity phenomenology for long, but it is likely that in
a few years we will look back at this paper as a paper that played
a valuable role getting us started in the right direction.
My personal opinion is that this sort of paper is important and
benefits particularly from publication in a prestigious journal.
I therefore recommend publication.

Reviewer #3:
Remarks to the Author:
I think the authors gave very thorough replies to all referees, and while I cannot speak for the other referees, the authors did adequately address the points raised in my own review. From my point of view, the replies to the other referees' comments are also adequate, and that the manuscript benefited from the scrutiny.

However, upon further review of the manuscript, I gave some more thought to the way the authors extracted data from reference 32 of the revised manuscript, and I think the authors should address a few points in this respect.

.) the size of the markers in Fig. 3 of Ref. 32 seem to be much larger than the error stated in that reference. In particular, the authors of Ref. 32 stated that the statistical error was 3×10^{-5} seconds. The size of the markers seems to be more on the order of 10^{-4} seconds. The authors said they zoomed in to extract the data point - probably from the tiny center of the markers that can be seen. This is an understandable assumption, but if it is not clearly stated that this small dot in the middle is the actual value, the authors may need to assume the full size of the markers as the measurement errors. On the other hand, that would slightly over-estimate the error provided in the old paper. One would see this if one calculates a reduced χ^2 value for the linear fit as I also indicate in my next comment.

.) comparing Table I of the manuscript with Fig. 3 of Ref. 32, it seems that there are significantly

more data points in that figure than in the table the authors provide. Did the authors include ALL the data points from Fig. 3 of that old paper? I am worried about that because there is of course some statistical uncertainty in the data points & not all of them agree with the fit within the errors of the measurements. This is as it should be. Statistically speaking only about two thirds of the data points should agree with the fit within the error. My worry is that the authors of the present manuscript might have extracted a subset of data points that might fit "too well". It would be interesting to see a reduce χ^2 value or similar to assess how well (or not) the extracted data points agree with the linear fit.

I think it would be prudent for the authors to address these concerns. Aside from that, I stand by my original assessment that I think that the paper in principle deserves publication in Nature Communications.

Response to referee

We appreciate all the referees' comments and suggestions, and we have modified our manuscript in accordance with the reports. Below we answer all of the referees' concerns in detail.

The referees' reports are reproduced verbatim and in full (in indented red sans-serif font), and our point-by-point responses are in non-indented black serif font. Changes that appear in the revised manuscript are in blue serif font. A document highlighting all the changes in the manuscript is appended to this response.

Response to Referee 1

> The authors have addressed all the issues I have raised in my first report and I think that the paper has improved in this respect. The approach and in particular the introduction of the exponent parameter α remains phenomenological, and deeper arguments on why one should adopt such a more general parametrization of the deformation parameter are not provided.
>
> Despite the significant improvement of the presentation, I still think that the paper should be more suitable for a more technical journal.

We thank the referee for their assessment of the improvement in presentation. The parameterization is indeed phenomenological but, unfortunately, in the absence of a full theory of quantum gravity to allow for a rigorous derivation of the scaling, we need to adopt this approach in order to provide experimental constraints on possible scaling exponents. This sentiment is also echoed by the report of Referee 2. Our hope is that obtaining such bounds experimentally could provide direction towards new theoretical models. Therefore, we believe that our work contains novel and significant contributions to the field and is of interest to a broad audience interested in tabletop tests of quantum gravity and is therefore suitable for publication in Nature Communications.

Response to Referee 2

> The authors have used my feedback and the feedback of other referees to significantly improve the manuscript. The model remains crude, but it is true that it is an attempt to model something that no one else has tried to model and needs to be modelled. The improvements made to the manuscript are such that now this crude model can me more efficiently received by the community. In summary I don't think this model will guide the relevant sub-area of quantum-gravity phenomenology for long, but it is likely that in a few years we will look back at this paper as a paper that played a valuable role getting us started in the right direction. My personal opinion is that this sort of paper is important and benefits particularly from publication in a prestigious journal. I therefore recommend publication.

We thank the referee for the positive assessment of the manuscript and the improvements made in this revision. We agree with the referee in the expectation that our work will serve as a first step that will guide the direction of this sub-area in the future.

Response to Referee 3

> I think the authors gave very thorough replies to all referees, and while I cannot speak for the other referees, the authors did adequately address the points raised in my own review. From my point of view, the replies to the other referees' comments are also adequate, and that the manuscript benefited from the scrutiny.

We thank the referee for the positive assessment of the revisions to the manuscript.

> However, upon further review of the manuscript, I gave some more thought to the way the authors extracted data from reference 32 of the revised manuscript, and I think the authors should address a few points in this respect.
>
> .) the size of the markers in Fig. 3 of Ref. 32 seem to be much larger than the error stated in that reference. In particular, the authors of Ref. 32 stated that the statistical error was 3×10^{-5} seconds. The size of the markers seems to be more on the order of 10^{-4} seconds. The authors said they zoomed in to extract the data point - probably from the tiny center of the markers that can be seen. This is an understandable assumption, but if it is not clearly

stated that this small dot in the middle is the actual value, the authors may need to assume the full size of the markers as the measurement errors. On the other hand, that would slightly over-estimate the error provided in the old paper. One would see this if one calculates a reduced χ^2 value for the linear fit as I also indicate in my next comment.

We appreciate the referee's concern regarding the error bars during data extraction. Previously, our model of calculating the error of extraction of the data was as follows: We plotted little triangles over the existing ones from Ref. [32] and changed the coordinates of the plotted triangles till there was visible deviation from the underlying triangle. The range of values of the plotted triangles that could not be discerned from the original triangle was the uncertainty of extraction.

However, as the referee correctly points out, Ref. [32] does not explicitly state that the small dot in the centre is the actual value. Therefore, we have updated the calculations to include the full size of the marker as the measurement errors as a conservative estimate and added text to clarify this point. We have made the following changes to the manuscript.

However, additional error arising from the datapoint extraction have to be accounted for. For this, we use the size of the markers as a conservative estimate. This leads to an error of 5×10^{-3} m^2 in the square of the amplitude and 10^{-4} s in the time period measurements.

We use the extracted data and account for both the sources of errors to perform a linear fit using the orthogonal distance regression method [36]. This fit yields a reduced χ^2 value of 0.07, which indicates that the extracted data points do indeed agree with the linear fit.

Due to the conservative estimate of the errors in the data extraction, the bounds on quantum gravity parameters are slightly modified. The bound on the parameters has now been modified from $\beta_0 N^{-\alpha} < 5 \times 10^{-4}$ to $\beta_0 N^{-\alpha} < 10^{-2}$, which implies that for $\beta_0 = 1$, the bound on the parameter α is modified from $\alpha > 0.12$ to $\alpha > 0.07$.

We also clarify that despite these conservative estimates, the conclusion of the manuscript, that is, that a macroscopic pendulum can provide the first positive bound on α assuming $\beta_0 = 1$, is unchanged. Furthermore, the bounds on the parameters obtained in the manuscript can be significantly improved with more precise experiments. We have added the following text to clarify his point.

We note that the current work is only a blueprint that uses experimental data from 1964 and such an analysis can be repeated with improved

experiments to determine tighter and more precise bounds.

We have also updated the Fig. 1 to reflect these changes in the bounds.

> .) comparing Table I of the manuscript with Fig. 3 of Ref. 32, it seems that there are significantly more data points in that figure than in the table the authors provide. Did the authors include ALL the data points from Fig. 3 of that old paper? I am worried about that because there is of course some statistical uncertainty in the data points and not all of them agree with the fit within the errors of the measurements. This is as it should be. Statistically speaking only about two thirds of the data points should agree with the fit within the error. My worry is that the authors of the present manuscript might have extracted a subset of data points that might fit "too well". It would be interesting to see a reduce χ^2 value or similar to assess how well (or not) the extracted data points agree with the linear fit.

We appreciate the thoroughness of the referee in analysing the data extraction and analysis of our work. Based on the referee's comments, we have included an extended discussion of the data extraction and analysis in our manuscript.

Firstly, we would like to clarify that Fig. 3 of Ref. [32] contains results from two experiments, one with a pendulum with a conventional suspension (triangular markers) and one with a cycloidal suspension (circular markers). In this light, the referee's question of whether we have included all data points includes two questions: One whether both experiments have been analysed and secondly whether all data points in a particular experiment have been included.

To answer the first question: in our analysis, we have only considered the experiment that uses the conventional suspension because such a system can be modelled by the analysis that we present in Sec. II A. In addition, unfortunately, Ref. [32] also does not contain enough experimental parameters about the cycloidal suspension to allow us to model it reliably.

We have added the following text to clarify this point in the manuscript.

> The figure contains data from two experiments: one using a conventional suspension of the pendulum and another using a cycloidal suspension. In this manuscript, we consider only the conventional suspension because such an experiment can be modelled by the calculations in Sec. II A.

To answer the second question of whether all points of the conventional suspension had been included: Due to an error in the extraction of the image from the paper, a

single datapoint (the one furthest away from the origin) was left out. We have now included that point in the analysis (and have updated Table 1 to reflect this). The conclusions remain unchanged.

In addition, we have added some more details about the procedure followed to extract the data in the Methods section.

> This data was extracted by magnifying from Fig. 3 of the paper, setting the figure as a plot background and plotting markers over the figure such that the plot points coincide exactly with the points in the background. The coordinates of the plotted points were recorded as the extracted data.

> I think it would be prudent for the authors to address these concerns. Aside from that, I stand by my original assessment that I think that the paper in principle deserves publication in Nature Communications.

We hope that the revised manuscript address all the points raised and thank the referee for the overall positive assessment of the manuscript.

The revised manuscript highlighting all additions and deletions is appended below. We trust that these modifications address the comments of the referees in full and hope that now the manuscript is suitable for publication in Nature Communications.

Reviewers' Comments:

Reviewer #3:
Remarks to the Author:
The authors have taken into account the points I raised regarding the data extraction to my satisfaction. While this had some impact on the overall results, as the authors pointed out, the main conclusions remain the same, and I agree with them, that carefully performed experiments would significantly improve on the bounds the authors extracted from older experiments.

I value the detailed response to my input! Extracting data from old experiments is tricky, but I am now confident that the manuscript now properly addresses this issue. My conclusion that I find the paper worthy of publication with Nature Communications still stands.
